# Autoimmune and immunoserological markers of COVID-19 pneumonia: Can they help in the assessment of disease severity

**DOI:** 10.3389/fmed.2022.934270

**Published:** 2022-08-29

**Authors:** Mihailo I. Stjepanovic, Maja R. Stojanovic, Sanja Stankovic, Jelena Cvejic, Sanja Dimic-Janjic, Spasoje Popevic, Ivana Buha, Slobodan Belic, Natasa Djurdjevic, Mirjana M. Stjepanovic, Dragana Jovanovic, Milica Stojkovic-Laloševic, Ivan Soldatovic, Branka Bonaci-Nikolic, Rada Miskovic

**Affiliations:** ^1^Faculty of Medicine, University of Belgrade, Belgrade, Serbia; ^2^Clinic for Pulmonology, University Clinical Center of Serbia, Belgrade, Serbia; ^3^Clinic of Allergy and Immunology, University Clinical Center of Serbia, Belgrade, Serbia; ^4^Faculty of Medical Sciences, University of Kragujevac, Kragujevac, Serbia; ^5^Center for Medical Biochemistry, University Clinical Center of Serbia, Belgrade, Serbia; ^6^Clinic of Psychiatry, University Medical Center Zvezdara, Belgrade, Serbia; ^7^Clinic of Gastroenterology and Hepatology, University Clinical Center of Serbia, Belgrade, Serbia; ^8^Institute of Medical Statistics and Informatic, Faculty of Medicine, University of Belgrade, Belgrade, Serbia

**Keywords:** COVID-19, autoimmunity, immunoglobulins, complement, pneumonia severity, anticardiolipin antibodies

## Abstract

**Background:**

Immune dysregulation and associated inefficient anti-viral immunity during Coronavirus Disease 2019 (COVID-19) can cause tissue and organ damage which shares many similarities with pathogenetic processes in systemic autoimmune diseases. In this study, we investigate wide range autoimmune and immunoserological markers in hospitalized patients with COVID-19.

**Methods:**

Study included 51 patients with confirmed Severe Acute Respiratory Syndrome Coronavirus 2 infection and hospitalized due to COVID-19 pneumonia. Wide spectrum autoantibodies associated with different autoimmune inflammatory rheumatic diseases were analyzed and correlated with clinical and laboratory features and pneumonia severity.

**Results:**

Antinuclear antibodies (ANA) positivity was found in 19.6%, anti-cardiolipin IgG antibodies (aCL IgG) in 15.7%, and anti-cardiolipin IgM antibodies (aCL IgM) in 7.8% of patients. Positive atypical x anti-neutrophil cytoplasmic antibodies (xANCA) were detected in 10.0% (all negative for Proteinase 3 and Myeloperoxidase) and rheumatoid factor was found in 8.2% of patients. None of tested autoantibodies were associated with disease or pneumonia severity, except for aCL IgG being significantly associated with higher pneumonia severity index (*p* = 0.036). Patients with reduced total serum IgG were more likely to require non-invasive mechanical ventilation (NIMV) (*p* < 0.0001). Serum concentrations of IgG (*p* = 0.003) and IgA (*p* = 0.032) were significantly lower in this group of patients. Higher total serum IgA (*p* = 0.009) was associated with mortality, with no difference in serum IgG (*p* = 0.115) or IgM (*p* = 0.175). Lethal outcome was associated with lower complement C4 (*p* = 0.013), while there was no difference in complement C3 concentration (*p* = 0.135).

**Conclusion:**

Increased autoimmune responses are present in moderate and severe COVID-19. Severe pneumonia is associated with the presence of aCL IgG, suggesting their role in disease pathogenesis. Evaluation of serum immunoglobulins and complement concentration could help assess the risk of non-invasive mechanical ventilation NIMV and poor outcome.

## Introduction

Viruses are known environmental factor contributing to the development of autoimmune diseases in susceptible individuals. Since the start of Coronavirus Disease 2019 (COVID-19) pandemic, increasing evidence suggests occurrence of autoimmune responses in patients with COVID-19 (1). Immune dysregulation during severe forms of COVID-19 leads to inefficient anti-viral immunity and increased immunopathology, causing tissue and organ damage (2). Vascular inflammation with associated endothelial injury and coagulopathy, characteristic for many autoimmune inflammatory rheumatic diseases (AIIRD), is present in severe COVID-19. Presence of several autoantibodies has been described in patients with COVID-19. Proposed mechanisms behind these autoimmune responses include molecular mimicry between Severe Acute Respiratory Syndrome Coronavirus-2 (SARS CoV-2) and human proteins, widespread tissue damage with increased release of autoantigens, neutrophil activation, formation of neutrophil extracellular traps and cytokine release (3). Whether this immune dysregulation persists after recovery, leading eventually to development of defined AIIRD is still unknown. Better characterization of autoimmune responses in patients with COVID-19 may provide new insights into the disease pathogenesis, prognosis, and treatment. We aimed to investigate the presence of autoimmune and immunoserological markers in patients with COVID-19 pneumonia and to test their correlation with selected clinical and laboratory features.

## Materials and methods

### Patients and study design

We included 51 patients with confirmed SARS-CoV-2 infection by real time reverse transcription–polymerase chain reaction (RT-PCR), hospitalized at the clinical ward at the Clinic for Pulmonology, University Clinical Center of Serbia in January 2021. Exclusion criteria were active malignant disease, previously confirmed AIIRD, thrombophilia, pregnancy, and use of relevant drugs at the time of COVID-19 diagnosis (corticosteroids, immunosuppressives, B-cell depleting drugs). Clinical, laboratory and radiological data were collected from medical records and clinical's health information system. Laboratory data taken closest to the hospital admission were analyzed. Immunological analysis was performed from blood samples collected on average 22.5 ± 10.9 days from symptoms onset.

Patients were treated according to the National Protocol for the treatment of COVID-19 infection. Antiviral treatment (favipiravir) was used during first 5–7 days since symptom onset in patients with risk factors for disease progression. Corticosteroid therapy (prednisone 0.5–1 mg/kg or dexamethasone 6 mg/day or methylprednisolone 1/2 mg/kg) was used in patients with moderate to severe/critical disease and clinical, laboratory or radiological signs of deterioration. Tocilizumab use was guided by the following criteria: CRP > 50 mg/l and IL-6 > 40 ng/l or three-fold increase of IL-6/CRP during last 48 h, and clinical deterioration. All patients received standard prophylactic doses of low-molecular weight heparin (LMWH), while therapeutical doses were used in those with suspected or confirmed thrombosis.

The severity of pneumonia was assessed using Pneumonia Severity Index (PSI), commonly used as mortality predictor in community acquired pneumonia, also found to perform well in COVID-19 pneumonia. Calculation of PSI score was done as previously described (4).

Systemic Inflammation Response Index (SII), a blood- cell count-derived inflammation index [(neutrophils × platelets)/lymphocytes] was calculated for each patient. According to the previous study, SII can be used as a prognostic biomarker in COVID-19, with an optimal cutoff value of 1,835 (5).

COVID-19 severity was assessed using National Institute of Health (NIH) severity categories: asymptomatic or presymptomatic infection, mild, moderate, severe, or critical illness. All patients in the study group were in moderate to critical severity category (6).

Patients were followed until the hospital discharge or death. Institutional review Board approved the study and waiver of informed consent.

### Detection of autoantibodies and immunoserological parameters

Serum samples of patients were analyzed for the presence of the following antibodies: antinuclear (ANA), antibodies to extractable nuclear antigens (ENA) specific for Sm, Sm/RNP, SSA, SSB, Jo-1, Scl-70, anti-neutrophil cytoplasmic antibodies (ANCA), anti-cardiolipin antibodies (aCL), anti-beta2-glycoprotein I antibodies (anti-β2-GPI), and rheumatoid factor (RF). Indirect immunofluorescence (IIF) assay on Hep-2 cells (Aesku Diagnostics, Germany) was used to detect and characterize ANA. ANA titer ≥ 1:80 was considered positive. Patients that were positive for ANA were tested for the presence of ENA by ELISA (Euroimmun, Germany). Presence of ANCA was determined using IIF, with titer ≥ 1:80 indicating positivity (Euroimmun, Germany). Samples showing positivity on IIF were additionally tested for ANCA specific for myeloperoxidase (MPO-ANCA) and proteinase 3 (PR3-ANCA) using enzyme-linked immunosorbent assay (ELISA) (Euroimmun, Germany). Anticardiolipin IgG/IgM and anti- β2-GPI IgG/IgM antibodies were measured using ELISA (Demeditec Diagnostics, Germany). We used manufacturer's recommended values for positive antibodies results (MPO-ANCA > 20 RU/ml, PR3-ANCA > 20 RU/ml, aCL IgG > 10 U/ml, aCL IgM > 7 U/ml, anti-β2-GPI IgG > 8 U/ml, anti-β2-GPI IgM > 8 U/ml). Serum concentrations of C3, C4, IgG, IgM, IgA, and RF assessed by nephelometric methods (Automatic Biochemistry analyzer Spin 200E-Spinreact), applying manufacturer's recommended reference values (C3 0.65–1.8 g/L; C4 0.1–0.4 g/L; IgG 7.0–16.0 g/L; IgM 0.4–2.3 g/L; IgA 0.7–4.0 g/L; RF > 20 IU/ml).

### Statistical analysis

Data were analyzed using the Statistical Package for the Social Sciences (SPSS) for Windows (version 26.0, Chicago, IL, USA). Quantitative variables were presented as mean and standard deviation (SD), or median and interquartile range (IQR) where appropriate. Categorical variables were presented as absolute and relative frequencies. Comparison of continuous variables was performed using Student's *t*-test or Mann-Whitney where appropriate. χ^2^ and Fisher's exact test were used to compare categorical variables. We considered *p*-value < 0.05 as statistically significant.

## Results

### Characteristics of the study group

Our study enrolled 51 patients with confirmed SARS CoV-2 infection. All patients were non-critically ill and treated at the clinical ward at the time of specimen collection. Important comorbidities were hypertension (58.8%), diabetes mellitus (21.6%), coronary artery disease (9.8%), malignant disease in remission (7.8%), chronic respiratory disease (13.7%), and chronic kidney disease (1.9%).

### Profile of autoantibodies in COVID-19 pneumonia

At least one autoantibody was found to be positive in 37.3% of patients. Most of them were positive for one-21.6%, while 15.7% were positive for 2 or more autoantibodies (Table 1). We found ANA positivity (titer ≥ 1:80) in 19.6% patients, with most specimens showing homogenous or speckled pattern on Hep2-cells. None of ANA positive patients showed ENA reactivity. All patients were tested for the presence of aCLs (aCL IgG and/or IgM), showing positivity in 21.6% (15.7%aCL IgG, 7.8%aCL IgM). Elevation of aCL was mild to moderate. The specimens that were positive for aCLs, were additionally tested for the presence of anti-β2-GPI IgG and IgM, showing positivity in only one patient that was positive for both aCL IgG and IgM. One patient that was positive to aCL IgG had thrombotic event during COVID-19 (acute myocardial infraction). Positive ANCA (atypical x ANCA in all cases) on IIF were detected in 10.0% of patients, but these specimens were negative for PR3-ANCA and MPO-ANCA. Positive RF was found in 8.2% of patients (Table 1).

**Table 1:** Prevalence and profile of autoantibodies in the study group.

**Autoantibody**	***n/N* (%)**
ANA	10/51 (19.6%)
**Titer:**	
1:80	6/10 (60%)
1:160	2/10 (20%)
1:320	1/10 (10%)
1:640	1/10 (10%)
ENA	0/10 (0%)
RF	4/49 (8.2%)
ANCA	5/50 (10%)
**Titer:**	
1:80	1/5 (20%)
1:160	3/5 (60%)
1:320	1/5 (20%)
aCL IgG	8/51 (15.7%)
aCL IgM	4/51 (7.8%)
aCL IgG and/or IgM	11/51 (21.6%)
Anti-beta2GP I IgG	1/11 (9.1%)
Anti-beta2GP I IgM	1/11 (9.1%)
**Number of positive autoantibodies:**	
**0**	32/51 (62.7%)
**1**	11/51 (21.6%)
**≥2**	8/51 (15.7%)
Any positive autoantibody	19 (37.3%)

ANA, antinuclear antibodies; ENA, extractable nuclear antigens; ANCA, anti-neutrophil cytoplasmic antibodies; aCL, anti-cardiolipin antibodies; anti-β2-GPI, anti-beta2-glycoprotein I antibodies; RF, rheumatoid factor.

### Immunoglobulin and complement level in COVID-19 pneumonia

Serum IgG was below the lower limit in 9 (17.7%), normal in 38 (74.5%) and higher than the upper limit of the reference range (7–16 g/l) in 4 (7.8%) patients. Serum IgM and IgA were within reference range in all patients. There were no significant changes in complement level in most patients. Low C4 and/or C3 was found in 3 (5.9%), and slightly elevated C4 in 5 (9.8%) patients (Table 2).

**Table 2:** Characteristics of patients according to the presence of autoantibodies.

**Characteristic**	**All patients** **(*N* = 51)**	**Autoantibody negative** **(*N* = 32)**	**Autoantibody positive** **(*N* = 19)**	***p-*value**
Sex (F/M)	21/30	10/22	11/8	0.062
Age (years)	65.7 ± 13.1	64.1 ± 14.9	68.4 ± 9.2	0.214
TE during COVID-19	8 (15.7%)	6 (18.8%)	2 (10.5%)	0.694
WBCs (×10^9^/l), med (IQR)	6.2 (4.3–10.1)	6.35 (4.45–11.1)	5.8 (4.3–8.8)	0.453
NETs (×10^9^/l, med (IQR)	4.5 (2.9–7.5)	5.35 (3.05–8.65)	4.0 (2.4–5.9)	0.094
LYMs (×10^9^/l), med (IQR)	0.8 (0.6–1.8)	0.8 (0.6–1.2)	0.8 (0.6–1.3)	0.837
RBCs (×10^12^/l), mean ± SD	4.35 ± 0.52	4.36 ± 0.52	4.35 ± 0.53	0.936
Hgb (g/l), mean ± SD	131.33 ± 15.75	132.91 ± 14.70	128.68 ± 17.47	0.360
PLTs (×10^9^/l), med (IQR)	174.0 (128–242)	180.5 (117–230)	172 (146–261)	0.311
Ferritin (ug/l), med (IQR)	688.5 (313–1.412)	687 (297–1,365)	694 (313–1,478)	0.712
D-dimer (mg/l), med (IQR)	0.98 (0.56–2.4)	1.0 (0.55–2.02)	0.72 (0.56–2.5)	0.770
PT (s), mean ± SD	12.83 ± 3.04	12.92 ± 3.38	12.69 ± 2.44	0.647
aPTT (s), mean ± SD	24.41 ± 5.12	24.95 ± 3.08	23.52 ± 7.42	0.340
PCT (ng/ml), med (IQR)	0.12 (0.07–0.25)	0.11 (0.07–0.22)	0.12 (0.07–0.3)	0.447
CRP (mg/l), med (IQR)	72 (44.2–103.0)	76.8 (38.4–105.0)	65.8 (50.0–100.0)	0.579
IL-6 (ng/l), med (IQR)	49.5 (22.9–116)	49.95 (21.45–89.7)	88.1 (24.9–141.0)	0.188
C3 (g/l), mean ± SD	1.26 ± 0.35	1.23 ± 0.35	1.30 ± 0.35	0.468
C4 (g/l), mean ± SD	0.27 ± 0.11	0.28 ± 0.12	0.26 ± 0.09	0.545
IgG (g/l), mean ± SD	10.05 ± 3.48	9.35 ± 3.63	11.2 ± 2.93	0.064
IgM (g/l), mean ± SD	1.1 ± 0.52	1.11 ± 0.58	1.07 ± 0.41	0.816
IgA (g/l), mean ± SD	2.55 ± 1.35	2.56 ± 1.42	2.53 ± 1.26	0.876
**Symptoms on admission**				
Cough	49 (96.1%)	30 (93.8%)	19 (100%)	0.523
Dyspnea	44/51 (86.3%)	26 (81.3%)	18 (94.7%)	0.236
Diarrhea	7/51 (13.7%)	7 (21.9%)	0	0.037
Headache	8/51 (16.0%)	4 (12.9%)	4 (21.1%)	0.459
Fever	46/51 (90.2%)	28 (87.5%)	18 (94.7%)	0.639
**NIH severity category**				
Mild	0	-	-	-
Moderate	19 (37.3%)	13 (40.6%)	6 (31.6%)	0.518
Severe and critical	32 (62.7%)	19 (59.4%)	13 (68.4%)	
PSI, mean ± SD	88.49 ± 30.21	89.13 ± 33.0	87.42 ± 25.65	0.845
≤ 90	26 (51.0%)	16 (50.0%)	10 (52.6%)	
>90	25 (49.0%)	16 (50.0%)	9 (47.4%)	0.856
SII score, med (IQR)	1.064 (500–2,041.2)	1,217.9 (486.8–2,086.5)	1,021.2 (500.6–1,626.7)	0.770
<1,835	37 (72.5%)	22 (68.8%)	15 (78.9%)	
≥1,835	14 (27.5%)	10 (31.3%)	4 (21.1)	0.430
Duration of symptoms (days)	22.5 ± 10.9	22.8 ± 13.6	21.1 ± 12.9	0.520
**Treatment**				
Corticosteroids	51 (100%)	-	-	-
Tocilizumab	9 (18.0%)	6 (19.4%)	3 (15.8%)	1.0
Favipiravir	5 (9.8%)	4 (12.5%)	1 (5.3%)	0.639
Oxygen	40 (80%)	29 (93.5%)	18 (94.7%)	1.0
NIMV	8 (16.0%)	4 (12.9%)	4 (21.1%)	0.459
LMWH	51 (100%)	-	-	-
**Outcome**				
Recovery	44 (86.3%)	28 (87.5%)	16 (84.2%)	1.0
Lethal outcome	7 (13.7%)	4 (12.5%)	3 (15.8%)	

TE, thrombotic events; WBCs, white blood cells; NETs, neutrophils; LYMs, lymphocytes; RBCs, red blood cells; Hgb, Hemoglobin; PLTs, platelets; PCT, procalcitonin; CRP, C-reactive protein.

### Correlation with clinical and laboratory parameters and pneumonia severity

First, we compared patients according to any autoantibody positivity. Patients with positive autoantibodies had higher serum IgG, and were more frequently women, but this difference was not statistically significant (*p* = 0.064, 0.062, respectively). We found no significant difference in the time interval between onset of COVID-19 symptoms and specimen collection for immunological analysis between autoantibody positive and negative patients (*p* = 0.520). More detailed demographic, clinical and laboratory characteristics of the study group, with comparison between autoantibody positive and negative patients are shown in Table 2.

ANA positivity wasn't associated with disease severity (*p* = 0.470), pneumonia severity (*p* = 0.291), outcome (*p* = 0.612), or markers of inflammation: CRP (*p* = 0.151), IL-6 (*p* = 0.652), ferritin (*p* = 0.112), SII (*p* = 0.722).

There were 21.6% patients who were positive for aCL IgG and/or IgM. Compared to aCL negative patients there was no difference in disease severity (*p* = 0.505), outcome (*p* = 0.635), or relevant laboratory parameters [aPTT (*p* = 0.492), D-dimer (*p* = 0.292), CRP (*p* = 0.423), ferritin (*p* = 0.202), IL-6 (*p* = 0.336), SII (*p* = 0.243)].There were 8 (15.7%) thrombotic events during COVID-19 (7 pulmonary thromboembolism, 1 acute myocardial infarction), but they were not associated with aCL positivity (*p* = 0.668). Anticardiolipin antibody positive patients had more frequently PSI score above 90 (72.7 vs. 42.5%, *p* = 0.076), and higher serum IgG (11.8 vs. 9.6, *p* = 0.062), but the difference wasn't statistically significant. Analysis of only aCL IgG positive patients showed significantly higher PSI score (103.7 vs. 85.6, *p* = 0.036) and total serum IgG (12.4 vs. 9.6, *p* = 0.032).

Considering low percentage of ANCA and RF positive patients, no further statistical analysis was performed.

**Figure 1:**
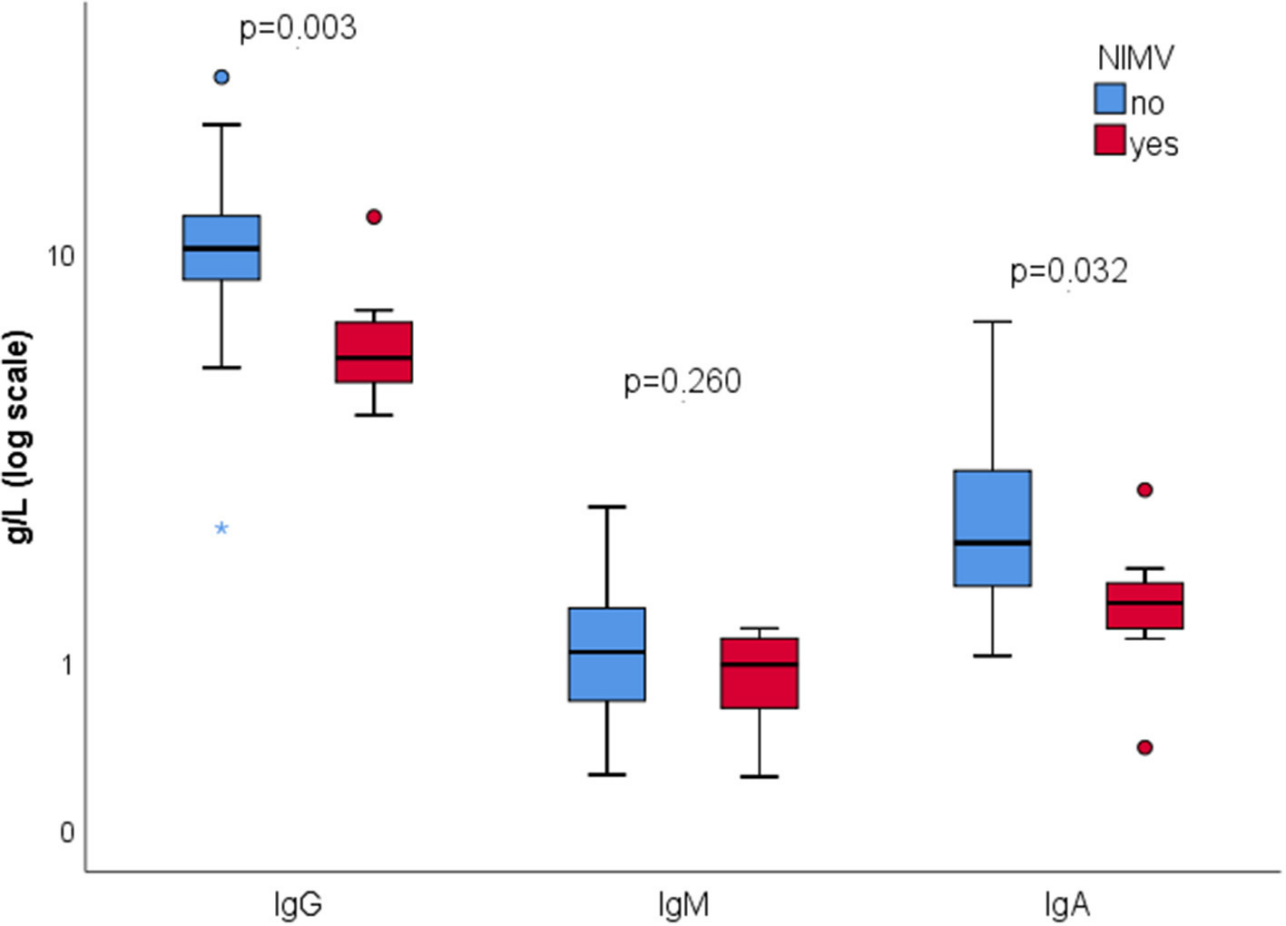
Total serum immunoglobulins concentration according to the use of NIMV (log scale).

We found no correlation between total IgG, IgA, IgM, and inflammation parameters CRP (*p* = 0.667, *p* = 0.452, *p* = 0.908, respectively), IL-6 (*p* = 0.072, *p* = 0.140, *p* = 0.261, respectively), ferritin (*p* = 0.422, *p* = 0.692, *p* = 0.886, respectively), SII (*p* = 0.993, *p* = 0.706, *p* = 0.063, respectively). Patients who experienced thromboembolic event during COVID-19 had significantly higher serum IgG (12.4 vs. 9.6, *p* = 0.033). There was no significant difference in immunoglobulin concentration according to the disease severity (*p* = 0.520, *p* = 0.177, *p* = 0.550, respectively). However, those with reduced total serum IgG were significantly more likely to require NIMV (OR = 39, 95% CI 5.3–283.8, *p* < 0.001). Comparison of immunoglobulin concentration showed lower serum IgG (*p* = 0.003) and IgA (*p* = 0.032), and no difference in serum IgM (*p* = 0.260) in patients on NIMV (Figure 1). All but one patient on NIMV survived.

We investigated weather severity of pneumonia is associated with different inflammatory and autoimmune markers (Table 3). Pneumonia severity was assessed using PSI. Depending on the PSI score, patients were stratified into two risk levels: low-risk (PSI score group I, II, and III, or PSI score ≤ 90) and high-risk (PSI score group IV and V or PSI score > 90). Patients in high-risk group were more frequently positive only for aCL IgG (28.0 vs. 3.8%, *p* = 0.024). Those on NIMV (112.0 ± 35.3 vs. 85.21 ± 26.8, *p* = 0.018) and NIH severe/critical category (99.7 ± 25.2 vs. 69.6 ± 28.9, *p* < 0.001) had significantly higher PSI score.

**Table 3:** Relevant comparisons according to the pneumonia severity.

**Characteristic**	**Low risk PSI score**	**High risk PSI score**	***p-*value**
WBCs (×10^9^/l), med, IQR	5.1 (3.9–7.5)	7.8 (5.8–13.8)	0.013
NETs (×10^9^/l), med, IQR	3.85 (2.6–5.7)	5.9 (4.1–11.4)	0.016
LYMs (×10^9^/l), med, IQR	0.8 (0.7–1.3)	0.8 (0.5–1.1)	0.306
PLTs (×10^9^/l), med, IQR	167 (128–225)	190 (126–253)	0.534
D-dimer (mg/l), med, IQR	0.71 (0.56–1.72)	1.7 (0.58–4.1)	0.099
Ferritin (ug/l), med, IQR	417.5 (282–771)	893.5 (589.5–1,604.5)	0.008
IL-6 (ng/l), med, IQR	40.55 (14.6–91.1)	58.2 (38.4–130.0)	0.101
PCT (ng/ml), med, IQR	0.09 (0.06–0.25)	0.12 (0.09–0.22)	0.496
CRP (mg/l), med, IQR	56.15 (24.7–80.9)	79.4 (65.0–107.0)	0.038
C3 (g/l), mean ± SD	1.34 ± 0.29	1.17 ± 0.38	0.073
C4 (g/l), mean ± SD	0.27 ± 0.12	0.26 ± 0.11	0.774
IgG (g/l), mean ± SD	10.4 ± 3.1	9.6 ± 3.8	0.411
IgM (g/l), mean ± SD	1.2 ± 0.5	0.9 ± 0.5	0.031
IgA (g/l), mean ± SD	2.5 ± 1.0	2.6 ± 1.6	0.687
SII, med, IQR	651.1 (485.0–1,337.0)	1,626.7 (627–2,379.7)	0.024
**NIH severity**			
Moderate	15 (57.7%)	4 (16.0%)	
Severe/critical	11 (42.3%)	21 (84.0%)	0.002
**Outcome**			
Recovery	24 (54.5%)	20 (45.5%)	
Lethal outcome	2 (28.6%)	5 (71.4%)	0.248
ANA	7 (26.9%)	3 (12.0%)	0.291
ANCA	3 (13.6%)	2 (8.0%)	0.654
aCL IgG and/or IgM	3 (11.5%)	8 (32%)	0.076
aCL IgG	1 (3.8%)	7 (28.0%)	0.024
RF	2 (8.0%)	2 (8.3%)	1.0

WBCs, white blood cells; NETs, neutrophils; LYMs, lymphocytes; RBCs, red blood cells; Hgb, Hemoglobin; PLTs, platelets; PCT, procalcitonin; CRP, C-reactive protein.

**Figure 2:**
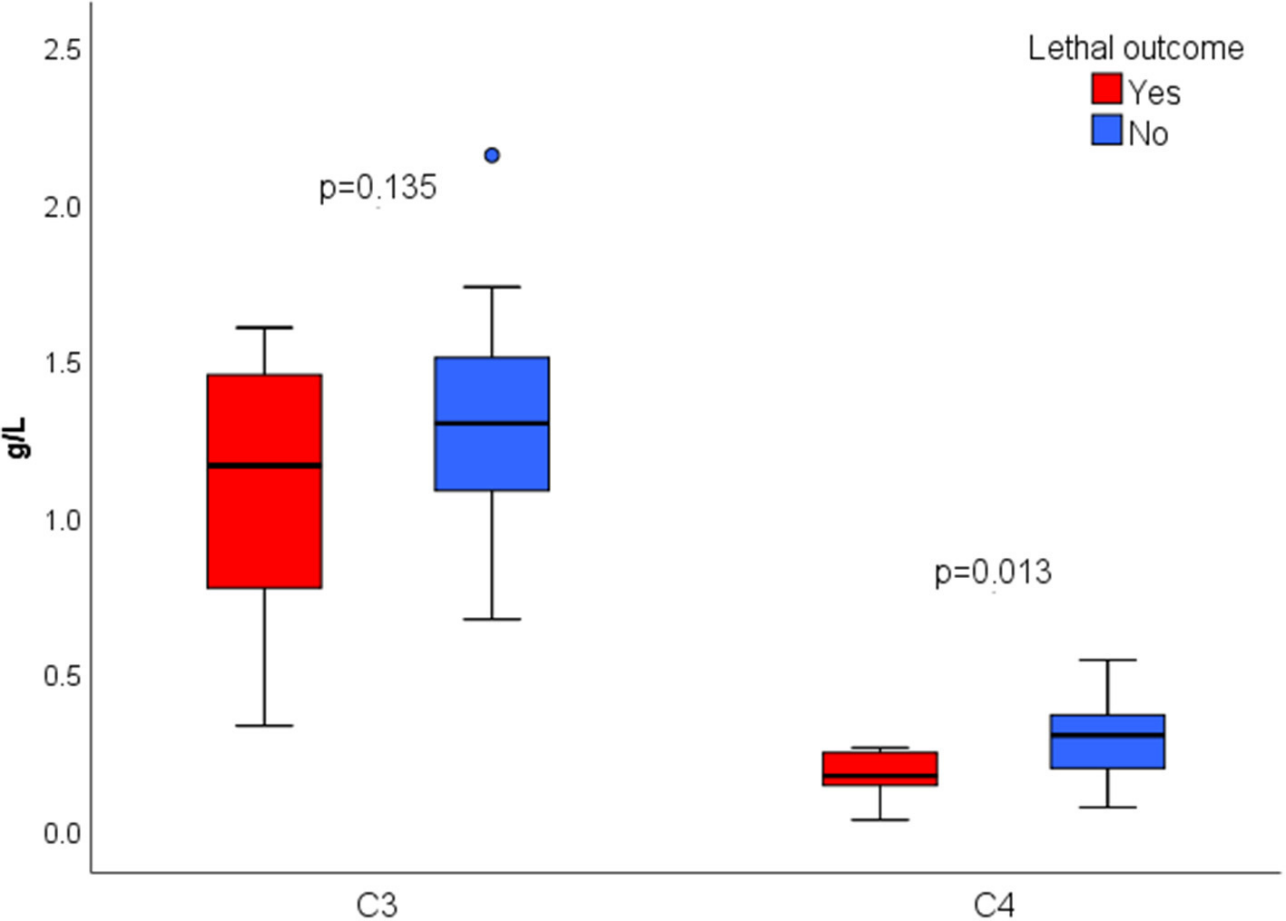
Serum C3 and C4 concentration according to the outcome.

**Figure 3:**
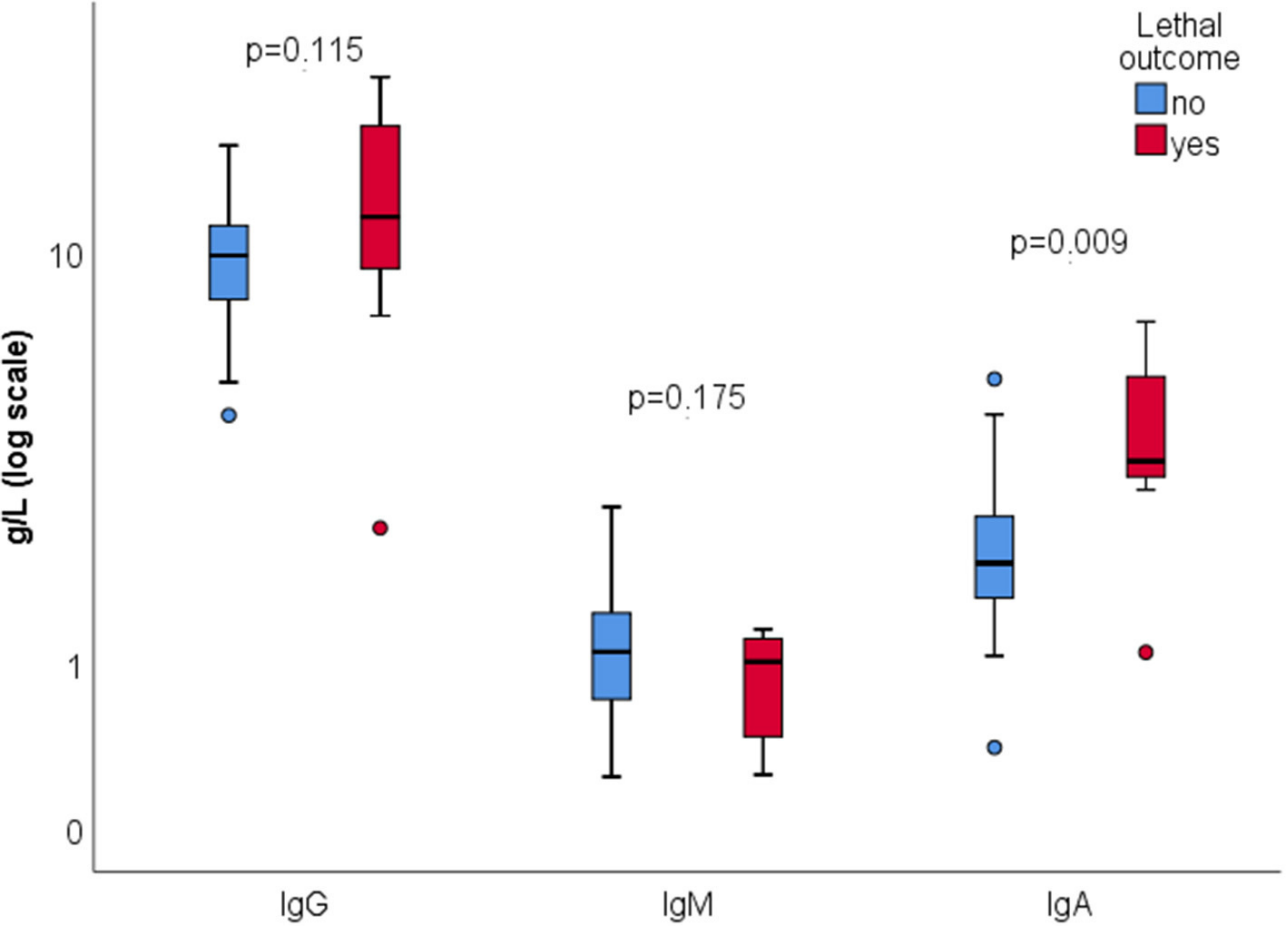
Total serum immunoglobulins according to the outcome.

There were 7 (13.7%) lethal outcomes. We found no association between the outcome and the presence of ANA (*p* = 0.612), aCL IgG and/or IgM (*p* = 0.635), RF (*p* = 0.092), or ANCA (*p* = 0.488). Lower C4 was found in deceased patients (0.17 vs. 0.28, *p* = 0.013), while C3 level wasn't significantly different (1.1 vs. 1.3, *p* = 0.135) (Figure 2). Higher serum IgA was associated with mortality, with no significant difference in serum IgG or IgM depending on the outcome (Figure 3).

## Discussion

Our study analyzed autoimmune and immunoserological responses in patients with COVID-19 pneumonia, and moderate to critical disease severity. Overall, autoantibody positivity was found in 37.3% of patients, with aCL IgG and/or IgM antibodies most frequently detected (21.6%). We found no association between thrombotic events during COVID-19 and aCL positivity. Presence of aCL IgG was associated with more severe pneumonia and higher total serum IgG. Patients with reduced total IgG were significantly more likely to require NIMV, while higher total IgG was associated with thromboembolic events during COVID-19. Deceased patients had significantly higher total IgA, and lower C4.

A literature review reported development over 15 different autoantibodies associated with SARS-Cov2 infection, mostly in patients with severe form of the disease (3). We found presence of ANA in 19.6% of patients, with most of them showing homogenous or speckled pattern on Hep2-cells. Strongly reactive ANA on IIF (≥1:160) was found in 40% of ANA positive patients. According to the literature data, positive ANA at 1:80 titer is found in 13.3% of general population, while only 5 and 3.3%, respectively, are found to be positive at titers 1:160 and 1:320 (7). None of ANA positive patients showed reactivity to ENA. Antinuclear antibody positivity has been associated with different infections probably reflecting transient activation of autoreactive B cells. An increased extrafollicular B cell activation, like one in autoimmune diseases is found in patients with severe COVID-19 (8). Moreover, significant neutrophilia and activation of neutrophils leading to the excessive production of NETs, another pathogenic feature of AIIRD, is present in severe COVID-19 (9). These similarities in pathogenesis of AIIRD and severe COVID-19 suggest that the presence of autoantibodies in COVID-19 patients is not just an epiphenomenon but might indicate loss of self-tolerance. Increased prevalence of ANA ranging from 25 to 57.5% in patients with COVID is reported by many studies (10–14). ANA titer and pattern were rarely reported. A study from Italy, reported 33% prevalence of ANA in titer 1:160–1:5,120, and mostly speckled and nucleolar pattern. Thereby, patients with unfavorable outcome were more frequently positive for ANA (14). However, an early pandemic study from China including only severe and critical COVID-19, found 50% ANA positivity using automatic immunoassay analyzer (12). Chang et al. reported positive correlation between ANA and development of anti-SARS Cov-2 IgG antibodies, linking development of ANA with antiviral responses. They also found that some autoantibodies are newly triggered by SARS Cov-2 infection additionally supporting break of self-tolerance during COVID-19 as an important pathogenic mechanism (13). In our study we found lower prevalence of ANA, which wasn't associated with COVID-19 or pneumonia severity, disease outcome or inflammatory markers. Lower rate of ANA positivity could be at least partially influenced by high prevalence of patients with moderate disease activity, who consisted 37.3% of the study group. Additional reasons for observed differences between studies include low number of patients in studies, differences in methodology of ANA determination and influence of genetic factors.

COVID-19 confers high risk for thrombotic events, both during infection and recovery period. Antiphospholipid antibodies (aPL) including aCL, anti-β2-GP I, and lupus anticoagulant (LA), are pathogenic antibodies targeting phospholipids and phospholipid-binding proteins. Transitory elevation of aPL has been reported in association with many infections and is considered an epiphenomenon of infection induced-immune dysregulation. Specifically, during COVID-19 the leakage of the surfactant from necrotic pneumocytes exposes the phospholipid-binding proteins to the immune system, triggering development of aPL (15). Although in most cases their presence is not associated with development of thrombosis, their exact role during and post-infectious period is not adequately explored. Anticardiolipin antibodies were most frequently detected antibodies in our patients (21.6%), dominantly of IgG isotype. Only one aCL positive patient was also anti-β2-GPI antibody positive (7.7%). Many studies have investigated aPL antibodies in COVID-19 with heterogenous results. The LA positivity was found in up to 90% (16). However, the LA test is functional assay, being influenced by multiple factors and should be interpreted with caution. The presence of aCL IgG is reported in 0–48%, and aCL IgM 0–21% (1, 17). Anti-β2-GPI antibodies are less frequent, with anti-β2-GPI IgM reported in 0–16%, and anti-β2-GPI IgG in 2–18.2% (17–20). Most of these studies included predominantly critically ill patients. The aPL level, when reported was lower than in patients with antiphospholipid syndrome (18, 20). In most cases there were no association with thrombosis, but aCL positivity had been associated with disease severity and poor outcome (1, 14, 21).

In our study group, aCL positivity wasn't associated with disease severity, outcome, or development of thrombotic events. Interesting finding was association between aCL IgG and severity of pneumonia, with significantly higher PSI score in aCL IgG positive patients. Conversely, patients with more severe pneumonia were more frequently positive for aCL IgG. This finding suggests a possible role of aCL IgG in pathogenesis of COVID-19 pneumonia, particularly in microvascular lung injury. It also raises a question whether severity of pneumonia and aCL IgG should be considered when deciding about LMWH therapy in a particular patient. As in previous studies we didn't find association between aPL and thrombosis during COVID-19. However, pathogenic potential of aPL should not be precluded considering that all hospitalized patients were on at least prophylactic doses of LMWH. Also, we didn't explore whether aPL positivity confers risk for development of thrombosis after hospital discharge, when in most cases anticoagulant therapy is suspended (1).

Considering that COVID-19 is often associated with neutrophilia, increased activation of neutrophils, formation of NETs, as well as with various vascular manifestations we sought to investigate presence of ANCA. Only 10.0% of patients were ANCA positive on IIF, mostly as atypical xANCA (titer 1:80–1:320), with none of them showing reactivity to vasculitis specific PR3 or MPO antigens. This is in concordance with previous study in which no patients showed ANCA reactivity (10, 14). Another study also didn't find elevated MPO-ANCA and PR3-ANCA, however they found BPI-ANCA in 6% of COVID-19 patients, supporting the role of NETs in disease pathogenesis and production of autoantibodies in severe COVID-19 (13).

Assessment of RF showed positivity in 8% of patients. Rheumatoid factor of IgM class recognizing Fc region of IgG, is commonly associated with rheumatoid arthritis, but is also frequent in number of systemic autoimmune diseases. Studies have shown that low-affinity polyreactive IgM RF may have beneficial role enhancing the clearance of immune complexes, while high-affinity RF, which is mostly associated with AIIRD, participates in stimulation of autoreactive lymphocytes and blood vessel deposition (22). Presence of RF was rarely investigated in patients with COVID-19. Similar to our results, study from China found RF IgM in 9.52% of patients. Moreover, RF IgM was associated with disease severity, and declined with the recovery (23). However, another study from China showed no RF positivity in severe COVID-19 (12). Whether RF is beneficial or harmful in COVID-19 requires further elucidation.

Protective effect of humoral immune response consists mainly through production of neutralizing antibodies, but also *via* antibody-dependent cellular cytotoxicity and immunomodulation (24). Worse disease severity and outcomes in viral infections are reported in patients with IgG and IgA deficiency (25, 26). We investigated the relationship between total serum immunoglobulins and different aspects of COVID-19 severity. Immunoglobulin level was not significantly different between NIH severity categories, or pneumonia severity except for mildly lower serum IgM in patients with more severe pneumonia. This finding probably reflects the fact that IgM antibodies occur early during the immune response and have shorter half-life. Further investigation showed that patients with reduced total serum IgG were significantly more likely to require NIMV. Also, there was significantly lower total IgA and IgG in patients on NIMV. However, the observed association could be confounded by a longer disease duration indicating that low IgG and IgA could be a marker of rapid catabolism of immunoglobulins during active infection and lymphocytes exhaustion. Whether this finding might be useful in predicting the need for intensive respiratory care in a particular patient, merits further exploration.

We also found higher serum IgA and IgG in deceased patients, but this difference was statistically significant only for IgA. Assessment of total serum immunoglobulins in patients with COVID-19 was infrequently done and revealed contradictory results. A study by Ali et al. found higher total IgA and IgA aPL (aCL, anti-β2-GPI), but not total IgG and IgG aPL antibodies in patients with severe illness (15). On the other hand, more recent study by Colkesen et al. found low serum IgA, IgG and IgG1 levels as independent risk factors for mortality in patients with COVID-19. Considering the abundance of ACE2 receptors in bronchial and gastrointestinal mucosa, strong stimulation of mucosal IgA is expected in patients with high viral load which is associated with severe disease. More studies, including serial immunoglobulin measurements are needed to elucidate the role of serum IgA in disease severity.

Overall, we speculate that these results could indicate existence of two disease phenotypes. One phenotype characterized with strong stimulation of immune system leading to heightened autoimmune responses and immunoglobulin level, benefiting from higher immunosuppression. Another phenotype would represent patients with initially low immunoglobulins, probably high viral load, and prolonged clinical course, possible benefiting from immunoglobulin replacement therapy.

Activation of complement in COVID-19 occurs *via* all three pathways (classical, lectin, and alternative) playing an important role in fighting against the virus. Increased and sustained complement activation, however, could aggravate diseases leading to poor outcome. There is histologic evidence in the lungs, kidney and the skin showing deposits of complement components and associated tissue injury (27). Complement activation participates in endothelial injury, coagulation activation and consequent thrombosis, starting, but not limited to the lungs (28). We assessed serum C3 and C4, which are routinely available indicators of complement activation, and found significantly lower C4 in deceased patients. Meta-analysis of 19 studies showed lower C3 and C4 concentration in patients with high disease activity and non-survivors. Most of included studies (18/19) originated from China with 10 of them conducted in the same hospital, possible influencing obtained results (27). Measurement of C3 and C4 could help identify patients at risk for lethal outcome but may also have therapeutical implications. Complement targeted therapies are currently being explored in COVID-19 (28).

The main limitations of the study are small sample size and lack of serial measurements. Relatively low number of patients prevents detection of all potential relations. Due to the study design and multiple comparisons our data require further investigation in larger clinical trials, before proving reliable in clinical practice.

The study represents thorough analysis of autoimmune and immunoserological markers in patients with COVID-19 pneumonia. We found increased autoimmune response not only in severe COVID-19 but also in those with moderate disease activity, with aCL IgG most frequently detected. Significant association between aCL IgG and severity of pneumonia implies possible causal role in disease pathogenesis and could have therapeutical implications. The association between low IgG and IgA and need for NIMV requires further investigation. Total serum IgA and C4 concentration should be investigated as possible predictors of mortality.

## Data availability statement

The raw data supporting the conclusions of this article will be made available by the authors, without undue reservation.

## Ethics statement

The studies involving human participants were reviewed and approved by Ethics Committee of the University Clinical Center of Serbia. Written informed consent for participation was not required for this study in accordance with the national legislation and the institutional requirements.

## Author contributions

MIS, MRS, BB-N, and RM done study concept and design and prepared the manuscript. MIS organized the database. IS performed the statistical analysis. MIS, MRS, SS, JC, SD-J, SP, IB, SB, ND, MMS, DJ, MS-L, BB-N, and RM performed the data collection and interpretation. All authors gave valuable interpretation of data, ensured that all aspects of the work are accurate, have been appropriately investigated and interpreted, contributed to manuscript revision, read, and approved the submitted version.

## Conflict of interest

The authors declare that the research was conducted in the absence of any commercial or financial relationships that could be construed as a potential conflict of interest. The reviewers IJ and VC declared a shared affiliation with the authors MRS, MIS, SD-J, SP, IB, DJ, MS-L, IS, BB-N, RM, and SS to the handling editor at the time of review.

## Publisher's note

All claims expressed in this article are solely those of the authors and do not necessarily represent those of their affiliated organizations, or those of the publisher, the editors and the reviewers. Any product that may be evaluated in this article, or claim that may be made by its manufacturer, is not guaranteed or endorsed by the publisher.
